# Breakfast Consumption May Improve Fasting Insulin, HOMA-IR, and HbA1c Levels in Predominately Low-Income, Hispanic Children 7–12 Years of Age

**DOI:** 10.3390/nu14112320

**Published:** 2022-05-31

**Authors:** Matthew R. Jeans, Sarvenaz Vandyousefi, Matthew J. Landry, Heather J. Leidy, Megan J. Gray, Molly S. Bray, Elizabeth M. Widen, Jaimie N. Davis

**Affiliations:** 1 Department of Nutritional Sciences, College of Natural Sciences, The University of Texas at Austin, Austin, TX 78723, USA; mjeans@utexas.edu (M.R.J.); heather.leidy@austin.utexas.edu (H.J.L.); mbray@austin.utexas.edu (M.S.B.); elizabeth.widen@austin.utexas.edu (E.M.W.); 2 Department of Medicine, Grossman Medical Center, VA New York Harbor Hospital, New York University, New York, NY 10016, USA; sarvenaz.vandyousefi@nyulangone.org; 3 Stanford Prevention Research Center, School of Medicine, Stanford University, Palo Alto, CA 94305, USA; matthewlandry@stanford.edu; 4 Department of Pediatrics, Dell Medical School, The University of Texas at Austin, Austin, TX 78723, USA; megan.gray@austin.utexas.edu

**Keywords:** breakfast, school-based intervention, glycemic control, nutrition, low-income, children

## Abstract

Children from low-income households and minority families have high cardiometabolic risk. Although breakfast consumption is known to improve cardiometabolic health in children, limited randomized control trials (RCT) have explored this association in low-income and racial/ethnic U.S. minority families. This study conducted secondary analyses from TX Sprouts, a school-based gardening, cooking, and nutrition education RCT, to examine the intervention effect on breakfast consumption and how changes in breakfast consumption impact cardiometabolic risk in predominately low-income, multi-ethnic children. TX Sprouts consisted of 16 schools (8 intervention; 8 control) in greater Austin, TX. A total of 18 lessons were taught, including topics on breakfast consumption benefits and choosing healthy food options at school. Children completed clinical measures (e.g., anthropometrics, body composition via bioelectrical impedance), and the number of breakfast occasions (BO) per week (at home and school) was captured via validated survey at baseline and post-intervention. Post-study—Baseline changes in breakfast consumption were used to categorize students as: maintainers (BO −1 to 1 day/week), decreasers (BO ≤−2 day/week), and increasers (BO ≥2 day/week). Optional fasting blood draws were performed on a subsample. Generalized weighted linear mixed modeling tested differences between intervention and control, with schools as random clusters. Analysis of covariance and linear regression examined changes in breakfast consumption on cardiometabolic outcomes, controlling for age, sex, race/ethnicity, free and reduced-price school meal participation (FRL), school site, breakfast location, physical activity, baseline cardiometabolic measures, and BMI z-score. This study included 1417 children (mean age 9 years; 53% male; 58% Hispanic, 63% FRL; breakfast consumption patterns: 63% maintainers, 16% decreasers, and 21% increasers). There was no intervention effect on changes in breakfast consumption. Compared to decreasers, increasers had an increase in insulin (−0.3 µIU/mL vs. +4.1 µIU/mL; *p* = 0.01) and a larger increase in HOMA-IR (+0.4 vs. +1.5; *p* < 0.01). Every one-day increase in breakfast consumption decreased fasting insulin by 0.44 µIU/mL, HOMA-IR by 0.11, and hemoglobin A1c by 0.01% (*p* ≤ 0.03). Increased breakfast consumption was linked to improved glucose control, suggesting breakfast can mitigate risk in a high-risk population. To better understand underlying mechanisms linking breakfast consumption to improved metabolic health, RCTs focusing on breakfast quality and timing are warranted.

## 1. Introduction

As of 2018, overweight (OW) and obesity (OB) affect 35.4% of children and adolescents 2–19 years of age in the United States [1]. Childhood OW/OB increases the risk of developing hypertension, type 2 diabetes, dyslipidemia, cardiovascular disease, and metabolic syndrome, as well as presents an increased risk of negative mental health outcomes, such as depression [2,3,4,5]. Dietary habits are modifiable behaviors that have been studied extensively to explain relationships with OW/OB and associated comorbidities [6,7]. Notably, breakfast consumption contributes to positive health outcomes through its role in energy maintenance and dietary regulation [8,9]. Longitudinal studies have shown that increased breakfast intake improves blood pressure, lipid panels, and glucose and insulin regulation, resulting in a lower risk of dyslipidemia and metabolic syndrome [10,11,12,13,14,15].

Despite breakfast consumption having stronger support and implications for improved cardiometabolic outcomes in children and adolescents, the U.S. Department of Health and Human Services reports that having breakfast on any given day decreases with age, dropping from 95.8% among those 2–5 years to 72.9% among those 12–19 years [16]. A decreasing trend in breakfast consumption was observed with age in all groups stratified by race and ethnicity and household income. Specifically, breakfast consumption was lower in Hispanic and non-Hispanic Black children and adolescents than their non-Hispanic White counterparts, and breakfast consumption decreased as household income decreased [16]. Non-Hispanic Black and Hispanic children and adolescents have high cardiometabolic risk relative to other races and ethnicities [1,17,18,19,20]. In addition, having low socioeconomic status is also associated with adverse health outcomes in childhood and later in life [21,22,23].

Breakfast consumption could be a modifiable dietary behavior that mitigates cardiometabolic risk in these populations, but causal relationships on health outcomes have yet to be elucidated. A recent systematic review that included eleven RCTs and eight intervention longitudinal studies on breakfast skipping and weight status in children and adolescents reported contradicting results [15]. Of those that examined breakfast skipping on OW/OB prevalence, the longitudinal studies reported higher adiposity in breakfast skippers while the RCTs reported no significant effects on weight or BMI [15]. However, a 12-week RCT not included in the review showed that high-protein breakfasts (35 g protein) prevented fat mass gains compared to normal-protein breakfasts (13 g protein), suggesting breakfast composition may play a prominent role in OW/OB risk [24]. Another systematic review of 37 observational studies supported that skipping breakfast is a marker of OW/OB risk and metabolic disease, but it could not establish causality, as 32 of the studies were cross-sectional and the remaining studies were longitudinal studies reporting cross-sectional data [11]. Most of the studies in these recent systematic reviews evaluating breakfast consumption in children and adolescents did not include high-risk populations, evaluate metabolic outcomes, or collect data on possible confounders, such as physical activity and breakfast location [11,15]. Robust experimental studies are needed in high-risk pediatric populations that evaluate comprehensive cardiometabolic profiles and account for potential confounders.

A previous cross-sectional study examined breakfast consumption in a predominately low-income, non-White pediatric population but showed no associations between breakfast consumption and several cardiometabolic outcomes [25]. Studies examining changes in breakfast consumption on cardiometabolic outcomes in a predominately low-income, non-White pediatric population are limited. Therefore, this secondary analysis from a RCT sought to evaluate (1) the impact of TX Sprouts, a gardening, cooking, and nutrition education intervention, on changes in breakfast consumption from pre-to-post intervention, and (2) the effect of changes in breakfast consumption on several anthropometric and metabolic outcomes in a predominately low-income, non-White pediatric population. We hypothesized that children in the TX Sprouts intervention compared to the control group would have increased breakfast consumption and that increased breakfast consumption would improve cardiometabolic outcomes.

## 2. Materials and Methods

### 2.1. Study Design

This secondary analysis from an experimental study used baseline and post-intervention data from TX Sprouts, a school-based cluster randomized controlled gardening, cooking, and nutrition intervention that was originally designed to increase fruit and vegetable intake and decrease sugar-sweetened beverage intake, obesity parameters, and blood pressure [26,27]. The complete methods of the TX Sprouts intervention have been described previously [28]. TX Sprouts recruited 3135 3rd–5th grade students and their parents from 16 greater Austin, TX, elementary schools. The inclusion criteria for schools were: (1) >50% proportion of Hispanic children, (2) >50% proportion of children enrolled in the free and reduced-price school meal participation, (3) location within 60 miles of the University of Texas at Austin campus, and (4) no pre-existing school garden or gardening program. The first 16 schools that met the criteria and agreed to participate were randomly assigned to (1) the intervention arm (*n* = 8 schools) or (2) the delayed intervention arm (*n* = 8 schools), serving as the control group. TX Sprouts was conducted over three waves, each lasting one school year, from 2016 to 2019. The intervention arm had three schools for the 2016–2017 (*n* = 6 total) and 2017–2018 (*n* = 6 total) school years and had two schools for the 2018–2019 school year (*n* = 4 total). Measures were collected at the beginning and end of each school year, approximately eight to nine months apart. This trial was registered at ClinicalTrials.gov (NCT02668744) (accessed on 9 May 2022).

### 2.2. TX Sprouts Intervention

The design and methodology of the TX Sprouts intervention has been described elsewhere [28]. In brief, TX Sprouts was a school-based gardening, cooking, and nutrition education intervention that incorporated the social ecological-transactional model into its core curriculum. This model rationalizes how processes within each level of ecology (e.g., family, school, community) exert reciprocal effects on one another to shape the course of child development [29].

While TX Sprouts was not designed to influence breakfast consumption, the programming could secondarily improve other dietary behaviors both within and outside the school environment. Full-time nutrition and garden educators taught 18 one-hour lessons to each 3rd–5th grade class throughout the school year during the school day, with lessons being adjusted for appropriate grade levels. Lesson topics included but were not limited to (1) whole foods vs. processed foods, (2) natural vs. added sugar, (3) fiber and whole grains, (4) food groups (e.g., role of protein, carbohydrates, fruits and vegetables), and (5) components of a healthy breakfast (e.g., fruits and vegetables, protein foods, and low-sugar, high-fiber carbohydrates). The curriculum on breakfast consumption focused on the health benefits of breakfast consumption (e.g., increased energy and metabolism, weight maintenance, controlled eating behaviors) and choosing nutritious breakfast options from the school cafeteria (e.g., white milk vs. chocolate milk; low-sugar cereal vs. high-sugar cereal; fresh fruit vs. fruit juice; limiting syrup, honey, and jam). Every lesson included either a garden taste-test or a cooking activity in addition to tastings of *aguas frescas*, which are infused waters with no added sugar. The curriculum was designed to be culturally tailored to Hispanic children, containing culturally relevant recipes, content, and activities. The control schools received a delayed intervention the following academic year and received the same protocol as those in the intervention arm as a delayed intervention in the following academic year.

### 2.3. Recruitment

Recruitment materials were available in both English and Spanish. Both parental consent and student assent were required for inclusion in the study. The study was conducted in accordance with the Declaration of Helsinki, and the Institutional Review Boards of The University of Texas at Austin (IRB#2014-11-0045) and all associated school district review boards approved all procedures pertaining to human subjects.

### 2.4. Survey Measurements

Students completed a survey at baseline and post-intervention (~8 months) of the following measures: demographics (i.e., age and sex), moderate to vigorous physical activity (MVPA), breakfast consumption, and typical weekday breakfast location. Table 1 presents the validated questions for MVPA, breakfast consumption, and typical weekday breakfast location [30,31]. The number of days each week (i.e., 0, 1–2, 3–4, and 5–7 days) breakfast items were usually consumed was captured via questions on the following: cereal (with milk), oatmeal, fruit, eggs/meat, breakfast sandwich, milk/yogurt, bread/bagel, pastries/sweets, and juice [30]. Free and reduced-price school meal participation, race, and ethnicity were reported by the parent/guardian. Individuals were categorized as Asian/Pacific Islander, Black/African American, Hispanic/Latino (including Mexican-American, Central American, and others), Native American/American Indian, non-Hispanic White, or “other” race and ethnicity.

### 2.5. Anthropometric and Physiological Measurements at Baseline and Post-Intervention

All participants were asked to remove footwear and heavy or layered clothing to obtain height (free-standing stadiometer to the nearest 0.1 cm; Seca, Birmingham, UK), body weight, and bioelectrical impedance (Tanita Body Fat Analyzer; Tanita Corporation of America Inc., Arlington Heights, IL, USA, model TBF 300). Participants were asked to collect clothing above the waist to measure waist circumference over skin using the National Health and Nutrition Examination Survey (NHANES) protocol in a private screening area [32]. BMI z-scores were calculated using the Centers for Disease Control and Prevention age- and sex-specific values [33]. Blood pressure was measured with an automated monitor (Omron, Schaumberg, IL, USA). In some cases, an adult cuff was used in place of a child cuff for proper fit to provide an accurate reading. All anthropometric and physiological parameters measures were taken once by trained staff.

### 2.6. Metabolic Measurements at Baseline and Post-Intervention

Fasting blood draws were optional, and those who opted to not participate in blood draws were able to participate in all other TX Sprouts activities and evaluations. Optional fasting blood draws were collected before the school day between 6:30 AM and 8 AM. Eligible students and their families received multiple reminders, via flyers and text message, about the optional blood draw and were instructed to arrive fasting, having nothing to eat or drink other than water after midnight. Certified phlebotomists and nurses with experience drawing blood in children with overweight and obesity collected blood samples in a private room at the schools. Students received a $20 incentive for participation in the blood draw. A free diabetes screening incentivized parents to have their children participate in the blood collection. Parents received their child’s fasting plasma glucose and glycated HbA1c values within two weeks of blood collection.

Whole blood was placed on ice directly following blood collection and transferred to the laboratory on the University of Texas at Austin campus, where fasting plasma glucose was measured using a HemoCue Glucose 201 analyzer (HemoCue America, Brea, CA, USA). HbA1c assays using DCA Vantage Analyzer (Siemens Medical Solutions, Malvern, PA, USA) were performed on whole blood. The remaining blood was centrifuged, aliquoted, and stored at −80 °C. Samples were transported on dry ice to Baylor College of Medicine to assess insulin, cholesterol, and triglycerides. Insulin was evaluated using an automated enzyme immunoassay system analyzer (Tosoh Bioscience, Inc., San Francisco, CA, USA). Homeostatic model assessment of insulin resistance (HOMA-IR) was calculated using the following formula: HOMA-IR = fasting glucose in mmol/l*fasting insulin in μU/mL/22.5 [34]. Total cholesterol, HDL cholesterol, and triglyceride levels were measured using Vitros chemistry DT slides (Ortho Clinical Diagnostics Inc., Rochester, NY, USA), and LDL cholesterol was calculated using the Friedwald equation [35].

### 2.7. Participants

**Figure 1:**
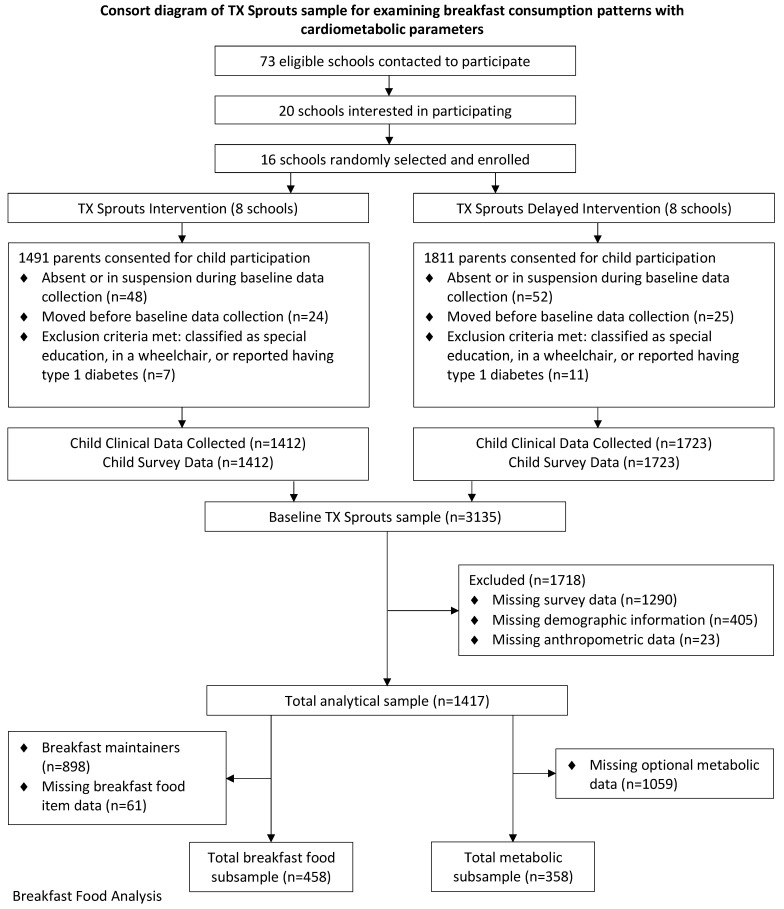
Consort diagram of TX Sprouts sample for examining breakfast consumption patterns with cardiometabolic parameters.

Figure 1 provides a detailed consort diagram showing the participant flow through the study. All 3rd–5th grade students in each school were eligible for the study (*n* = 4239). Both student assent and parental consent were obtained for 3302 students to participate in the TX Sprouts intervention. Of those, clinical data were collected on 3135 students. Characteristics of the total TX Sprouts population are published elsewhere [28]. This study analyzed a subsample from the TX Sprouts intervention to perform a complete-case analysis on students who had anthropometric measures as well as on breakfast data collected via survey, which were collected only in the last two waves of the RCT. In addition to the exclusion criteria for the design of the intervention outlined in Figure 1, students were excluded from analyses for missing demographic data (*n* = 405), breakfast and physical activity survey data (*n* = 1290), and anthropometric data (*n* = 23) at baseline and/or post-intervention. The total analytical sample was 1417 students for the intervention’s effect on breakfast consumption and changes in breakfast consumption on anthropometric parameters. Subsequent analyses were performed on decreasers and increasers who had completed survey data on breakfast food items to potentially explain mechanisms for changes in health outcomes (*n* = 458). Subsequent analyses were performed on metabolic parameters from a fasting blood draw, which was an optional measurement. Since a larger-than-expected proportion of students were found to have prediabetes, based on the American Diabetes Association definition [36] (fasting plasma glucose of 100–125 mg/dL), a glycolated hemoglobin A1c (HbA1c) measurement was added in the last two waves, which contributed to the lower number of those who had complete metabolic panels at baseline and post-intervention (*n* = 358).

### 2.8. Statistical Analysis

All study data were managed in Research Electronic Data Capture (REDCap) at The University of Texas at Austin. Changes in breakfast consumption between baseline and post-intervention were analyzed both as a continuous and categorical predictor. As a continuous measure, change in the number of breakfast occasions was the difference between post-intervention and baseline measures (ranging −7 to 7). As a categorical measure, change in the number of breakfast occasions between post-intervention and baseline was defined in three groups: (1) maintainers, those who had minimal change in breakfast consumption (change in breakfast occasions ranging −1 to 1); (2) decreasers, those who had negative change in breakfast consumption (change in breakfast occasions ≤−2); and (3) increasers, those who had positive change in breakfast consumption (change in breakfast occasions ≥2).

For demographic data (i.e., age, sex, race, and ethnicity) and breakfast consumption, generalized weighted linear mixed models (GLMM) with the identity link were used to test differences between the intervention and the control estimates, with schools as random clusters. GLMM with the identity link were used to compute *p*-values of the continuous variables, and GLMM with the logit link were used to compute *p*-values of the categorical variables. Following the null results from the impact of the intervention, changes in breakfast consumption, independent of the intervention group, were examined. First, summary statistics were performed to describe sociodemographic characteristics between breakfast consumption patterns. Chi-square (*X*^2^) tests and univariate analyses of variance were performed to examine differences in study participant characteristics between breakfast consumption patterns. ANCOVAs were performed to examine relationships between changes in breakfast consumption on cardiometabolic parameters, which were followed by a Bonferroni post hoc analysis. Linear regression examined the change in cardiometabolic parameters with every one-day increase in breakfast occasions. *X*^2^ tests were performed in secondary analyses of breakfast food items between decreasers and increasers who had completed survey data on breakfast food items to potentially explain mechanisms for changes in health outcomes (*n* = 458). These models were adjusted for sex, age, race and ethnicity, free and reduced-price school meal participation, school site, typical weekday breakfast location, physical activity, baseline cardiometabolic measure, and BMI z-score (except for models with BMI percentile, waist circumference, and body fat percentage as the outcome). Breakfast location (i.e., home, school, and other) was included as a covariate due to a previous study in this population that showed breakfast composition to be different between the home and school environments [37]. BMI percentile, waist circumference, diastolic blood pressure, fasting insulin, HOMA-IR, total cholesterol, HDL cholesterol, non-HDL cholesterol, LDL cholesterol, and triglycerides were transformed for normality. Data were analyzed using StataSE (Version 17.0, StataCorp, 2021, College Station, TX, USA).

## 3. Results

There were no significant differences in demographic measures between the intervention and control groups in this analytic sample (data not shown). There was also no significant intervention effect on breakfast consumption (i.e., the number of breakfast occasions) (intervention: +0.3 ±2.0 vs. control: +0.2 ±2.0 (mean ± SD); *p* = 0.79). Therefore, the remaining results report changes in breakfast consumption patterns, independent of intervention group, on post-intervention cardiometabolic outcomes.

Demographic characteristics of the sample between breakfast consumption categories are presented in Table 2. The study population was 53% male and had an average age of 9.3 years at baseline. The sample was 58% Hispanic, and 63% of children participated in the free and reduced-price school meal participation at school. Approximately 44% of children had OW/OB. Most students were classified as breakfast maintainers (63%), followed by breakfast increasers (21%) and breakfast decreasers (16%). Participation in the free and reduced-price school meal participation differed by breakfast consumption patterns, with higher participation among breakfast maintainers and increasers than decreasers. No other differences in sociodemographic characteristics (i.e., age, sex, race and ethnicity), typical weekday breakfast location, or BMI categories were observed between breakfast consumption patterns.

The relationships between changes in breakfast consumption and cardiometabolic outcomes via ANCOVA are presented in Table 3. No differences were observed in adiposity measures between breakfast consumption patterns. However, compared to breakfast decreasers, breakfast increasers had lower fasting insulin (21.0 µIU/mL vs. 18.7 µIU/mL, respectively; *p* = 0.01) and HOMA-IR (5.2 vs. 4.5, respectively; *p* = 0.006). The relationships between changes in breakfast consumption and cardiometabolic outcomes estimated with linear regression are presented in Table 4. For every one-day increase in breakfast consumption, there was a decrease in fasting insulin (β = −0.44; *p* = 0.003), HOMA-IR (β = −0.11; *p* = 0.002), and HbA1c (β = −0.01; *p* = 0.03). Differences in the frequencies of breakfast food items consumed weekly by breakfast consumption patterns are presented in Table 5. However, there were no significant differences observed for breakfast food items consumed between the breakfast consumption patterns.

## 4. Discussion

This is the first experimental study to examine the effects of a gardening, cooking, and nutrition education intervention on breakfast consumption, and to report findings between changes in breakfast consumption and cardiometabolic parameters, in a predominately low-income, non-White pediatric population. This study found no main intervention effect on changes in breakfast consumption from pre-to-post intervention and showed no relationships between changes in breakfast consumption on anthropometric parameters or blood pressure. However, increased breakfast consumption was associated with decreased fasting insulin, HOMA-IR, and HbA1c levels in a subsample of participants with an optional fasting blood draw. The frequencies of typically consumed breakfast food items were also examined in a subsequent analysis, but no significant differences were observed between breakfast consumption change patterns with the foods consumed. These findings highlight the impact of breakfast consumption, independent of food composition, on improving glycemic control in a high-risk population. Early behavioral interventions targeting increased breakfast consumption in high-risk children could be beneficial to decrease metabolic risk and potentially prevent further disease onset into adulthood.

Increased breakfast consumption resulted in increased glucose control (i.e., fasting insulin, HOMA-IR, and HbA1c) in a predominately low-income non-White population. Given Hispanic and non-Hispanic Black children and adolescents have been reported to have higher fasting insulin, HOMA-IR levels, and type 2 diabetes prevalence than their non-Hispanic White counterparts [19,38], targeting increased breakfast consumption in these populations could mitigate risk. Furthermore, children in this study were predominately low-income, and low socioeconomic status in childhood is associated with increased risk for impaired fasting glucose and diseases caused by insulin resistance, such as metabolic syndrome and type 2 diabetes, in adulthood [22,39]. The present study showed a modest decrease in fasting insulin levels with increased breakfast consumption but a notable increase in fasting insulin levels with decreased breakfast consumption (–0.3 µIU/mL vs. +4.1 µIU/mL, respectively), suggesting increased breakfast consumption may facilitate insulin level homeostasis. In addition, linear regression showed that for every additional day breakfast was consumed in a one-week period from pre-to-post intervention, fasting insulin decreased 0.44 µIU/mL. These results translate to as much as a 3.08 µIU/mL decrease in insulin if consumed daily for one week. Though HOMA-IR increased in both groups, it was significantly lower among breakfast increasers than breakfast decreasers (+0.4 vs. +1.5, respectively) and had an effect size of as much as −0.77. Many studies that have evaluated breakfast consumption on cardiometabolic outcomes in pediatric populations have not assessed fasting insulin, HOMA-IR, and HbA1c, but similar results have been shown in other studies in adolescents and adults. A longitudinal study consisting of a national sample of Australian children (9–15 years old) investigated associations between breakfast consumption and cardiometabolic outcomes over a 20-year period and found that those who did not consume breakfast at baseline and 20 years later had higher fasting insulin and HOMA-IR [40]. Marlatt and colleagues reported that breakfast consumption in 367 adolescents (11–18 years old) was also associated with lower fasting insulin and HOMA-IR levels [41]. The findings from this study suggest that increasing regular breakfast consumption could mitigate cardiometabolic risk, particularly in low-income, non-White populations, but the mechanism for this improvement warrants further exploration.

The frequency and timing of breakfast can impact glucose metabolism in many ways. A two-week randomized crossover trial of ten lean women found no difference in fasting insulin levels between consuming breakfast and omitting breakfast, but postprandial insulin levels were higher when omitting breakfast compared to consuming breakfast [42]. While this study is often cited and contrary to the findings from the present study, two weeks may not be long enough to affect fasting insulin levels. Furthermore, that study only included lean adults, and glucose metabolism is variable to body composition, with leaner individuals having greater insulin sensitivity. However, Arslanian and colleagues reported greater insulin resistance in adolescents than adults, despite similar levels of adiposity and glycemic control, and this could be a result of the more substantial effect obesity has on insulin sensitivity in youth compared to adults [43,44]. Due to the relatively high obesity prevalence in this pediatric population, the present study suggests that increased breakfast consumption could lead to lower insulin levels in a high-risk pediatric population.

The results on HbA1c levels showed that every one-day increase in breakfast consumption decreased HbA1c 0.01%, which could translate into an effect size of as much as 0.07%. While this observation may have limited clinical significance, HbA1c has been regarded as a reliable biomarker for the onset of type 2 diabetes and other diseases [45]. Khaw and colleagues reported that an HbA1c increase of 1% is associated with a 30% increase in all-cause mortality and a 40% increase in cardiovascular or ischemic heart disease mortality, and an HbA1c reduction of 0.2% could lower mortality by 10% in those who have type 2 diabetes [46]. Similar results have also been reported in studies evaluating the relationships between type and frequency of breakfast consumption with HbA1c. A cross-sectional study that included 5316 young adults (20–39 years old) reported that, compared to those who skipped breakfast, those who consumed breakfast were less likely to have elevated HbA1c, regardless of whether breakfast consisted of only ready-to-eat cereal (RTEC) or “other” foods [12]. Another study suggested that children who consumed breakfast daily had favorable type 2 diabetes risk profiles (i.e., fasting insulin, glucose, and HbA1c), especially in those who consumed high-fiber breakfast cereal [47]. Conversely, more robust experimental studies are needed to replicate these results to elucidate the mechanism for HbA1c improvement.

Other research posits that high caloric intake earlier in the day may influence glucose metabolism. Jakubowicz and colleagues conducted a 12-week randomized, open-label, parallel-arm study comparing two isocaloric diets, one with a 700-calorie breakfast and 200-calorie dinner and the other vice versa. The diet with a higher caloric intake at breakfast reduced body weight, waist circumference, and fasting glucose and insulin levels more than in the high-calorie dinner group [48]. Chowdhury and colleagues performed an RCT as a follow-up study to Jakubowicz et al. to examine causal links between breakfast habits and energy balance in adults with obesity for over 12 weeks [48,49]. The results concluded that those randomized to consume at least 700 calories before 11AM had greater insulin sensitivity than those who fasted until noon, but there was no impact of the intervention on body weight, which could be due to fasting participants compensating for the lack of morning energy intake [49]. Another RCT had similar results, showing that a high-energy breakfast compared to dinner had no change in BMI, waist circumference, and adiposity between groups, but resulted in reductions in fasting plasma glucose, insulin, and HOMA-IR [50]. The present study also showed associations with glucose control but no associations with adiposity parameters. The USDA reports that both Hispanic and non-Hispanic Black children (6–11 years old) have higher energy consumption at breakfast than their non-Hispanic White counterparts (21% and 20% vs. 17%, respectively) [51]. Data on energy intake were not captured in the current study, but 66% of the study population was Hispanic or non-Hispanic Black. One mechanism that explains the glucose metabolism benefits observed from higher caloric intake at breakfast is that of circadian rhythms [52]. Glucose tolerance is lower and skeletal muscle fatty acid oxidation is higher in the morning, so shifting food intake to earlier in the day in alignment with those rhythms has been shown to improve glycemic control in adults [53,54]. Thus, high energy consumption at breakfast in this pediatric population could be a plausible rationale for the observed improvements in metabolic parameters without associated improvement in adiposity parameters, but more robust, controlled studies are needed to validate these results in children.

Examining the duration for which regular breakfast consumption occurs has yielded inconsistent results on OW/OB prevalence and underscored the need for more robust, longitudinal studies. Data from the National Longitudinal Study of Adolescent Health showed that U.S. adolescents who consumed breakfast regularly during both adolescence (11–18 years of age) and young adulthood (18–26 years of age) were less likely to have OB compared to those who had irregular breakfast consumption at both time points [55]. Regular breakfast consumption over a prolonged period may be needed to affect adiposity. The present study lasted approximately eight months and consisted of a high-risk population, being predominately low-income and non-White, with 44% of children having OW/OB. While this study noted increased breakfast consumption in 21% of children, the duration from which breakfast consumption increased is unknown. It could be that increased breakfast consumption did not occur until a relatively short time before post-intervention measures were collected. Thus, there was not enough time to influence adiposity outcomes in a high-risk population. In addition, much of the literature that has shown inverse associations between breakfast consumption and adiposity or weight status has been cross-sectional [8,11,15]. Monzani and colleagues included 37 articles in their review of breakfast intake of weight outcomes, with only 5 of those being longitudinal studies, and a total of 6 studies showed null relationships between breakfast consumption and OW/OB status [11]. Ricotti and colleagues only examined RCTs (*n* = 11) and intervention longitudinal trials (*n* = 5) and still reported conflicting results between breakfast consumption and adiposity parameters, with null relationships observed in four studies, and a negative impact observed in one study [15]. These systematic reviews highlight discrepancies in breakfast consumption on adiposity parameters but emphasize the need for more experimental and longitudinal studies to elucidate these relationships in children.

The primary objective of the TX Sprouts intervention was to improve dietary intake (i.e., fruit and vegetable consumption) and cardiometabolic health [28]. The intervention increased vegetable intake [26,27], but the present study showed no impact on breakfast consumption. While it was not a primary focus of the intervention curriculum, one of the eighteen lessons in the intervention encouraged breakfast consumption and taught (1) the healthy components of a breakfast meal, (2) the health benefits of breakfast consumption, and (3) choosing healthy breakfast options from the school cafeteria. Other school-based interventions and RCTs have targeted breakfast consumption through alternative methodologies, such as School Breakfast Program participation, breakfast in the classroom initiatives, school-based health promotion programs, and breakfast promotion campaigns [56,57,58,59,60,61,62]. Many of these were implemented for one year or longer and encouraged breakfast intake through incorporating breakfast-specific nutrition education in classrooms, evaluating breakfast policies, and providing training courses for teachers at primary school to promote healthy lifestyle choices to their students. The null effects of the intervention on breakfast consumption could be due to one breakfast-specific lesson over the span of one school year being an insufficient amount of instruction to increase breakfast intake, particularly since it was taught earlier in the intervention. Even so, both a school-based intervention and RCT reported increased breakfast consumption at school led to students consuming a second breakfast, possibly contributing to higher OB prevalence [58,61]. School-based programs have been successful at increasing breakfast consumption, but initiatives implementing policy-based interventions, such as breakfast in the classroom, need to examine the impact of double breakfast consumption on health outcomes and determine whether or not students should be allowed to receive a second breakfast meal.

In addition to breakfast consumption, composition or quality of breakfast intake could have an additive or deleterious effect on health outcomes. Breakfast increasers had more frequent consumption of cereal (with milk) and milk/yogurt than breakfast decreasers, but these differences were not statistically different (*p* = 0.07 and *p* = 0.06, respectively). Even so, children and adolescents who regularly consume RTEC breakfasts have more nutritious intake at breakfast due to higher consumption of whole grains and milk/dairy products that are normally consumed with them [63,64,65,66,67]. RTEC and milk/dairy products are primary contributors to protein, whole grain, and fiber consumption at breakfast in children and adolescents [68]. High protein intake at breakfast (35 g or 40% of energy) has been shown to improve weight management, glucose metabolism, and satiety and appetite control throughout the day [24,69,70,71,72]. Similarly, high-fiber (28 g) breakfast consumption decreased several adiposity parameters compared to low-fiber (3 g) breakfast consumption [73]. Cereal breakfast consumers, compared to skippers and non-cereal consumers, had higher carbohydrate, total sugars, fiber, and micronutrient intake overall, but there were no differences in several anthropometric parameters [66,74]. A cross-sectional study that examined breakfast consumption in this cohort reported null findings on cardiometabolic outcomes but also noted breakfast consumers had higher total carbohydrate, total sugar, and added sugar consumption compared to skippers [25]. The higher consumption of cereal with milk and milk/dairy products observed in this study could partially explain the metabolic benefits received from increased breakfast consumption. However, the potentially higher intakes of sugars and refined carbohydrates could negatively affect anthropometric measures, and a high amount of protein (35 g) or fiber (28 g) may be required to see intended positive effects on weight outcomes.

The current study had limitations to consider. First, breakfast consumption was captured via self-report and had no specific parameters regarding energy or time of breakfast consumption. The parameters included were broad categories, limiting the ability to determine specific mechanisms behind energy intake and dietary composition, rendering it unrepresentative of a typical diet at the individual level. In addition, data on reasons for skipping breakfast were not collected, so the interpretation of results on the intervention effect on changes in breakfast consumption was limited. The analysis also assumed one breakfast meal was consumed for each day any breakfast consumption was reported in the survey; however, some children may have had double breakfast occasions. However, the survey instrument used to capture breakfast consumption and foods typically consumed was validated [30]. While MVPA can have a profound effect on glycemia and insulin resistance and was controlled for in the models, the survey measure on MVPA was limited to one day prior to data collection and is not indicative of daily MVPA. However, it was adapted from a gardening and physical activity intervention, Texas!Go!Eat!Grow! [31]. Furthermore, no measures were collected between baseline and post-intervention, so the analyses cannot consider the duration of increased or decreased breakfast consumption in interpreting the associations with cardiometabolic outcomes. Linear regression was performed to show the effect for every one-unit increase in breakfast consumption on cardiometabolic outcomes. The study population was predominately low-income and Hispanic, so stratification of race and ethnicity in the analyses could not be achieved, and the results obtained may not be generalizable to other pediatric populations. Conversely, the study highlights a relationship that may improve metabolic outcomes in this high-risk homogenous population.

## 5. Conclusions

This study showed that increased breakfast consumption has protective effects on fasting insulin, HOMA-IR, and HbA1c in a predominately low-income, non-White population. However, changes in breakfast consumption did not affect anthropometric parameters. While this study posits that breakfast consumption is an effective dietary behavior to improve glycemia in a high-risk pediatric population, future experimental studies are needed to replicate these data and elucidate mechanisms for these relationships.

## Figures and Tables

**Table 1:** Key survey variables of interest.

Variable	Survey Question	Response Options
Breakfast Consumption [30]	How many school days each week do you typically eat breakfast?	0–5 (0 = None, 1 = 1 school day, 2 = 2 school days, 3 = 3 school days, 4 = 4 school days, 5 = 5 school days)
	How many weekend days each week do you typically eat breakfast?	0–2 (0 = None, 1 = 1 weekend day, 2 = 2 weekend days)
Breakfast Weekday Location [30]	Where do you typically eat breakfast during the school week? (only select one option)	0–4 (0 = At home (by myself), 1 = At home (with family), 2 = At school (in cafeteria), 3 = At school (in class), 4 = Other
Moderate to Vigorous Physical Activity [31]	Yesterday, did you do any moderate to vigorous (very active) physical activities for about 30 min (about the time you get to eat lunch at school) DURING THE DAY? (list of 12 examples)	0, 1 (0 = No, 1 = Yes)

**Table 2:** Sociodemographic and physical characteristics of participants by breakfast consumption patterns.

Variable	Total	Maintainers	Decreasers	Increasers	*p*-Value ^a^
Sample size (n)	1417	898	220	299	
Sex (M), *n* (%)	753 (53.1)	475 (33.5)	117 (8.3)	161 (11.4)	0.96
Age (years), mean ± SD	9.3 ± 0.9	9.3 ± 0.9	9.3 ± 0.9	9.3 ± 0.9	0.66
Race and Ethnicity, *n* (%)					0.09
Hispanic	825 (58.2)	502 (35.4)	140 (9.9)	183 (12.9)	
Non-Hispanic White	405 (28.6)	283 (20.0)	51 (3.6)	71 (5.0)	
Non-Hispanic Black	113 (8.0)	69 (4.9)	17 (1.2)	27 (1.9)	
Other ^b^	74 (5.2)	44 (3.1)	12 (0.8)	18 (1.3)	
Free/Reduced-Price School Meal, *n* (%)	888 (62.7)	516 (36.4)	167 (11.8)	205 (14.5)	<0.001
Breakfast Weekday Location, *n*(%)					0.16
Home	734 (51.8)	487 (54.2)	101 (45.9)	146 (48.8)	
School	628 (44.3)	379 (42.2)	110 (50.0)	139 (46.5)	
Other	55 (3.9)	32 (3.6)	9 (4.1)	14 (4.7)	
BMI categories, ^c^ *n* (%)					0.13
Underweight	38 (2.7)	26 (1.8)	2 (0.1)	10 (0.7)	
Normal	760 (53.6)	503 (35.5)	108 (7.6)	149 (10.5)	
Overweight	254 (17.9)	150 (10.6)	45 (3.2)	59 (4.2)	
Obese	365 (25.8)	219 (15.5)	65 (4.6)	81 (5.7)	

^a^ Significance set at *p* < 0.05. ^b^ Native American/American Indian, Asian/Pacific Islander, more than one race, and “other”. ^c^ BMI categories were based on BMI percentiles using Centers for Disease Control age- and sex-specific values. Underweight was classified as < 5th percentile, normal weight was classified as 5th percentile to < 85th percentile, overweight was classified as 85th percentile to < 95th percentile, and obese was classified as ≥ 95th percentile.

**Table 3:** ANCOVA ^a^ models examining anthropometric and metabolic parameters of participants by breakfast consumption ^b^.

	Maintainers			Decreasers (D)			Increasers (I)				
Variable	Baseline Mean ± SD	Post Mean ± SD	Absolute Change Mean ± SD	Baseline Mean ± SD	Post Mean ± SD	Absolute Change Mean ± SD	Baseline Mean ± SD	Post Mean ± SD	Absolute Change Mean ± SD	*p*-Value ^c^	Bonferroni
Anthropometric parameters ^d^											
Sample size (n)	898	898	898	220	220	220	299	299	299		
Waist circumference (cm)	69.5 ± 11.7	70.8 ± 12.0	1.4 ± 3.7	71.8 ± 12.2	73.3 ± 12.8	1.5 ± 4.2	70.7 ± 12.6	72.2 ± 13.0	1.5 ± 3.4	0.50	--
Total body fat (%)	24.8 ± 8.5	24.3 ± 8.8	−0.5 ± 2.6	26.7 ± 8.8	26.4 ± 9.2	−0.3 ± 3.0	26.3 ± 9.1	25.8 ± 9.3	−0.5 ± 3.0	0.64	--
BMI^e^ percentile	66.7 ± 30.3	65.8 ± 30.8	0.8 ± 9.3	72.4 ± 28.5	72.0 ± 29.1	0.4 ± 9.1	71.5 ± 28.2	70.5 ± 28.4	1.1 ± 7.9	0.88	--
Physiological parameters ^f^											
Systolic blood pressure (mmHg)	102.1 ± 11.3	102.4 ± 11.2	0.2 ± 11.7	103.7 ± 11.5	104.4 ± 10.5	0.7 ± 12.5	103.1 ± 13.4	103.3 ± 12.1	0.2 ± 12.8	0.44	--
Diastolic blood pressure (mmHg)	66.3 ± 9.1	67.2 ± 9.4	0.9 ± 11.1	67.4 ± 9.3	67.0 ± 7.3	−0.4 ± 10.4	67.3 ± 11.6	67.1 ± 10.0	−0.2 ± 11.5	0.43	--
Metabolic parameters ^g^											
Sample size (*n*)	229	229	229	59	59	59	70	70	70		
Fasting glucose (mg/dL) ^h^	89.8 ± 8.9	96.1 ± 9.4	6.3 ± 11.3	88.6 ± 9.0	96.6 ± 9.9	8.0 ± 11.4	88.1 ± 7.8	94.5 ± 9.4	6.3 ± 10.7	0.07	--
Insulin (µIU/mL) ^i^	15.3 ± 11.0	15.8 ± 10.3	0.6 ± 8.3	16.9 ± 12.3	21.0 ± 23.9	4.1 ± 15.5	19.0 ± 17.8	18.7 ± 18.4	−0.3 ± 13.6	0.01	D vs. I, 0.01
HOMA-IR ^j^	3.4 ± 2.5	3.8 ± 2.6	0.4 ± 2.2	3.7 ± 2.7	5.2 ± 6.8	1.5 ± 4.9	4.1 ± 3.7	4.5 ± 4.9	0.4 ± 3.5	0.007	D vs. I, 0.006
Cholesterol (mg/dL) ^k^	149.7 ± 23.2	146.7 ± 24.4	−3.0 ± 18.6	150.4 ± 28.6	150.9 ± 26.2	0.5 ± 17.2	156.5 ± 31.9	149.3 ± 30.1	−7.2 ± 14.4	0.36	--
HDL (mg/dL)	48.9 ± 9.9	50.0 ± 10.8	1.1 ± 6.6	45.0 ± 10.9	46.6 ± 10.9	1.6 ± 4.9	48.6 ± 10.4	47.7 ± 10.1	−0.9 ± 6.0	0.25	--
Non-HDL (mg/dL)	100.8 ± 21.5	96.8 ± 22.0	−4.0 ± 15.0	105.5 ± 25.9	104.4 ± 24.2	−1.1 ± 15.2	108.0 ± 29.9	101.7 ± 29.1	−6.3 ± 12.6	0.36	--
LDL (mg/dL)	83.2 ± 18.1	79.1 ± 19.9	−4.1 ± 14.7	84.5 ± 22.0	82.8 ± 21.7	−1.7 ± 14.8	87.6 ± 29.0	83.1 ± 28.3	−4.5 ± 11.8	0.44	--
Triglycerides (mg/dL) ^l^	88.7 ± 41.1	88.5 ± 46.2	−0.2 ± 37.8	105.2 ± 49.2	108.3 ± 54.1	3.0 ± 37.9	101.6 ± 50.1	93.1 ± 41.9	−8.5 ± 41.7	0.48	--
HbA1c (%)	5.2 ± 0.3	5.3 ± 0.3	0.02 ± 0.2	5.2 ± 0.3	5.3 ± 0.3	0.06 ± 0.2	5.2 ± 0.2	5.2 ± 0.2	0.01 ± 0.2	0.12	--

^a^ ANCOVA: analysis of covariance. ^b^ All values represent mean ± SD. ^c^ Significance set at *p* < 0.05. ^d^ ANCOVA models for anthropometric outcomes adjusted for age, sex, race and ethnicity, free/reduced-price school meal participation, school site, breakfast location, physical activity, and baseline measure. ^e^ BMI: body mass index. ^f^ ANCOVA models for anthropometric outcomes adjusted for age, sex, race and ethnicity, free/reduced-price school meal participation, school site, breakfast location, physical activity, baseline measure, and BMI z-score. ^g^ ANCOVA models for metabolic parameters adjusted for age, sex, race, ethnicity, free/reduced-price school meal participation, school site, breakfast location, physical activity, baseline measure, and BMI z-score. ^h^ To convert mg/dL glucose to mmol/L, multiply mg/dL by 0.0555. ^i^ To convert µIU/mL insulin to pmol/L, multiply µIU/mL by 6.945. ^j^ HOMA-IR: homeostatic model assessment of insulin resistance. ^k^ To convert mg/dL cholesterol to mmol/L, multiply mg/dL by 0.0259. ^l^ To convert mg/dL triglycerides to mmol/L, multiply by mg/dL by 0.0113.

**Table 4:** Regression models examining anthropometric and metabolic parameters of participants by changes in breakfast consumption ^a^.

Variable	Β	95% CI	*p*-Value ^b^
Anthropometric ^c^ parameters ^c^ (*n* = 1417)			
Waist circumference (cm)	0.01	(−0.09, 0.11)	0.88
Total body fat (%)	−0.02	(−0.09, 0.06)	0.67
BMI percentile	−0.09	(−0.33, 0.15)	0.34
Systolic blood pressure (mmHg)	−0.08	(−0.33, 0.18)	0.56
Diastolic blood pressure (mmHg)	−0.01	(−0.22, 0.24)	0.91
Metabolic parameters ^d^ (*n* = 358)			
Fasting glucose (mg/dL) ^e^	−0.42	(−0.93, 0.08)	0.10
Insulin (µIU/mL) ^f^	−0.44	(−1.04, 0.16)	0.003
HOMA-IR ^g^	−0.11	(−0.29, 0.06)	0.002
Cholesterol (mg/dL) ^h^	−0.02	(−0.96, 0.91)	0.72
HDL (mg/dL)	−0.23	(−0.58, 0.11)	0.22
Non-HDL (mg/dL)	0.14	(−0.64, 0.92)	0.93
LDL (mg/dL)	0.21	(−0.55, 0.97)	0.99
Triglycerides (mg/dL) ^i^	−0.35	(−2.35, 1.66)	0.93
HbA1c (%)	−0.01	(−0.02, −0.001)	0.03

^a^ All values represent mean ± SD. ^b^ Significance set at *p* < 0.05. ^c^ Regression models for anthropometric outcomes adjusted for age, sex, race and ethnicity, free/reduced-price school meal participation, school site, breakfast location, physical activity, baseline measure, and BMI z-score (for blood pressure models only). ^d^ Regression models for metabolic parameters adjusted for age, sex, race and ethnicity, free/reduced-price school meal participation status, school site, breakfast location, physical activity, baseline measure, and BMI z-score. ^e^ To convert mg/dL glucose to mmol/L, multiply mg/dL by 0.0555. ^f^ To convert µIU/mL insulin to pmol/L, multiple µIU/mL by 6.945. ^g^ HOMA-IR: homeostatic model assessment of insulin resistance. ^h^ To convert mg/dL cholesterol to mmol/L, multiply mg/dL by 0.0259. ^i^ To convert mg/dL triglycerides to mmol/L, multiply by mg/dL by 0.0113.

**Table 5:** Chi-square examining consumption frequencies of breakfast food items in predominately low-income children by breakfast consumption patterns.

	Decreasers (*n* = 198)			Increasers (*n* = 260)			
Survey Items	Response *n* (%)			Response *n* (%)			*p*-Value ^a^
	0–2 x/Week	3–4 x/Week	5–7 x/Week	0–2 x/Week	3–4 x/Week	5–7 x/Week	
Cereal (with milk)	143 (72.2%)	29 (14.7%)	26 (13.1%)	161 (61.9%)	53 (20.4%)	46 (17.7%)	0.07
Oatmeal	176 (88.9%)	16 (8.1%)	6 (3.0%)	228 (87.7%)	19 (7.3%)	13 (5.0%)	0.56
Fruit	117 (59.0%)	50 (25.3%)	31 (15.7%)	145 (55.7%)	61 (23.5%)	54 (20.8%)	0.38
Eggs/meat	132 (66.7%)	46 (23.2%)	20 (10.1%)	178 (68.4%)	55 (21.2%)	27 (10.4%)	0.50
Breakfast sandwich	164 (82.9%)	27 (13.6%)	7 (3.5%)	224 (86.1%)	20 (7.7%)	16 (6.2%)	0.06
Milk/yogurt	146 (73.7%)	23 (11.6%)	29 (14.7%)	190 (73.1%)	46 (17.7%)	24 (9.2%)	0.06
Bread/bagel	159 (80.3%)	26 (13.1%)	13 (6.6%)	211 (81.1%)	29 (11.2%)	20 (7.7%)	0.75
Pastries/sweets	157 (79.3%)	21 (10.6%)	20 (10.1%)	201 (77.3%)	30 (11.5%)	29 (11.2%)	0.88
Juice ^b^	132 (66.6%)	37 (18.7%)	29 (14.7%)	161 (61.9%)	45 (17.3%)	54 (20.8%)	0.24

^a^ Significance set at *p* < 0.05. ^b^ Type of juice (e.g., 100% fruit juice, etc.) was not captured via survey.

## Data Availability

The data that support the findings of this study are available from the corresponding author (J.N.D.) upon request.
